# Blue care: a systematic review of blue space interventions for health and wellbeing

**DOI:** 10.1093/heapro/day103

**Published:** 2018-12-18

**Authors:** Easkey Britton, Gesche Kindermann, Christine Domegan, Caitriona Carlin

**Affiliations:** 1 Whitaker Institute, National University of Ireland – Galway, Galway, Ireland; 2 Applied Ecology Unit, Centre for Environmental Science, National University of Ireland – Galway, Galway, Ireland; 3 J.E. Cairnes School of Business & Economics, National University of Ireland – Galway, Galway, Ireland

**Keywords:** blue space, blue health, nature-based therapy, intervention, systematic review

## Abstract

There is increasing interest in the potential use of outdoor water environments, or blue space, in the promotion of human health and wellbeing. However, therapeutic nature-based practices are currently outpacing policy and the evidence base for health or wellbeing benefits of therapeutic interventions within blue space has not been systematically assessed. This systematic review aims to address the gap in understanding the impacts of blue space within existing interventions for targeted individuals. A systematic review was carried out, searching Google Scholar, SCOPUS, PubMed, etc. through to August 2017. Only blue space interventions were included that were specifically designed and structured with a therapeutic purpose for individuals with a defined need and did not include nature-based promotion projects or casual recreation in the outdoors. Thirty-three studies met the inclusion criteria and were assessed. Overall, the studies suggest that blue care can have direct benefit for health, especially mental health and psycho-social wellbeing. The majority of papers found a positive or weak association between blue care and health and wellbeing indicators. There was also some evidence for greater social connectedness during and after interventions, but results were inconsistent and mixed across studies with very few findings for physical health. This is the first systematic review of the literature on blue care. In summary, it has been shown that mental health, especially psycho-social wellbeing, can be improved with investment in blue spaces. Key areas for future research include improving understanding of the mechanisms through which blue care can improve public health promotion.

## INTRODUCTION

Most of the earth's surface is covered by water, and most of the human body is composed of water—two facts illustrating the critical linkages between water, health and ecosystems. (WHO, 2017)

As the above statement from the World Health Organisation (WHO) highlights, water environments are essential to promote health. Nevertheless, global evidence of disconnect and detachment from our natural surroundings is growing as the world’s ecosystems increasingly come under threat from human pressures (Levin and Poe, 2017), with economic and social goals attained at the cost of future health (Kite-Powell, 2008). Freshwater, coastal and marine ecosystems have been identified as suffering more rapid degradation and biodiversity loss than any other ecosystems (MEA, 2005; Whitmee *et al.*, 2015). With over one-third of the world’s population living around coastal ecosystems (Neumann *et al.*, 2015) attention has more recently begun to focus on blue space and promoting human health (Domegan *et al.*, 2016; Grellier *et al.*, 2017). Within academia there is growing interdisciplinary interest in and recognition for the benefits provided by specific water environments, or ‘blue space’ (Korpela *et al.*, 2010; Depledge *et al.*, 2013; Wheeler *et al.*, 2015; Bell *et al.*, 2015, 2018). This systematic review builds on this evidence to look at the use of blue space in therapeutic interventions for the promotion of health and wellbeing.

The WHO define health as ‘a state of complete physical, mental and social wellbeing and not merely the absence of disease or infirmity’ yet public and political discourse is preoccupied with disease (Yach *et al.*, 2004; Kim *et al.*, 2013; South, 2015). Within the UK, and indeed globally, the growing interest in the therapeutic potential of nature-based interventions at a policy-level (Bragg and Atkins, 2016) seems to be driven by a global health crisis, in particular the rise of non-communicable diseases (Kickbusch, 2015). The WHO reported that 88% of deaths in the European region were caused by non-communicable diseases (WHO, 2016) such as obesity, type 2 diabetes and mental illness often attributed to increasing sedentary lifestyles, poor diet, an ageing population and social isolation in developed nations (Bragg and Atkins, 2016). This crisis is further aggravated by overburdened and underfunded public health care systems (Kaplan and Porter, 2011; Kirwan *et al.*, 2017). The issue of mental health is especially acute, with a rising suicide rate and lack of funding for services highlighted in the UK (Mental Health Taskforce, 2016). Public health authorities are beginning to recognize the importance of proximity to, and contact with, natural environments ‘as an upstream health promotion intervention for populations’ [(Maller *et al.*, 2006), p. 45]. Although public health interventions delivered at the individual or community level can be equally successful in changing the behaviour of a large population (Sniehotta *et al.*, 2017) they cannot be seen in isolation from other environmental factors which could exert a greater influence on behaviour change (Graham and White, 2016). Challenging these preoccupations is one of the key goals of an emerging number of research initiatives, collaborative research projects such as ‘NEAR Health’ and ‘Blue Health’ are building an evidence base that will begin to qualify how important natural environments, and uniquely aquatic environments, are for human health and wellbeing.

### The link between nature, health and wellbeing

While there is some conceptual ambiguity of terms such as ‘blue space’, ‘health’ and ‘wellbeing’ (Bragg and Atkins, 2016), a systematic review requires definitions of terminology. Blue space could be described as a ‘threshold concept’ (Meyer and Land, 2003) and is often assumed under the umbrella concept of green space or green infrastructure where the assumption is that these spaces will ‘improve environmental conditions and therefore citizens’ health and quality of life’ (EC, 2016). Blue space is largely defined in the academic literature to include all visible outdoor surface waters (White *et al.*, 2016; Grellier *et al.*, 2017), however, blue space is sometimes still subsumed under ‘green space’, in particular riparian areas (Haeffner *et al.*, 2017). Foley and Kistemann’s [(Foley and Kistemann, 2015), p. 157] definition emphasizes the health enabling qualities, ‘where water is at the centre of a range of environments with identifiable potential for the promotion of human wellbeing’. In this paper, blue space is used to refer to all visible, outdoor, natural surface waters with potential for the promotion of human health and wellbeing. This excludes outdoor swimming pools, garden ponds and fountains, however, it can include modified and artificially constructed spaces that still contain natural surface water such as a canals, dammed lakes or urban streams/rivers. It is evident that there is much overlap between blue and green spaces, however, authors have argued that blue spaces offer very different sensory experiences and are used in different ways with different outcomes and benefits that are often overlooked and remain poorly understood (Haeffner *et al.*, 2017).

A term increasingly used in environmental policy and management is ‘nature-based solutions’ (NBS). NBS are defined by the European Commission (EC) as ‘instruments inspired by nature and using the properties and functions of ecosystems to enhance ecosystem services and multiple health benefits’ [(Haase *et al.*, 2017), p. 42]. The concept of nature-based therapy is defined by the Green Care Coalition in the UK as, ‘nature-based therapy or treatment interventions specifically designed, structured and facilitated for individuals with a defined need’ [(Sempik and Bragg, 2016), p. 100]. These terms are emerging and evolving and encompass any intervention that uses or learns from nature to improve health or manage illness. The term ‘blue care’ is used in this paper to refer to blue space interventions (BSI), pre-designed activities or programmes (typically physical) in a natural water setting, targeting individuals to manage illness, promote or restore health and/or wellbeing for that group.

There is a growing body of international literature exploring how engagement with nature can assist both in promoting and restoring health (Maller *et al.*, 2006; Coombes *et al.*, 2010; Lovell *et al.*, 2014; Sandifer *et al.*, 2015). However, a specific focus on blue space for health and wellbeing has only emerged in more recent years (Foley and Kistemann, 2015). To date, only one systematic review has focused exclusively on blue space (Gascon *et al.*, 2017), and one scoping review on urban, freshwater blue space (Völker and Kistemann, 2011). Gascon *et al.* (Gascon *et al.*, 2017) synthesized current epidemiological evidence from 36 quantitative studies on the health benefits of blue spaces. The review found that overall there were potential health benefits of living near or deliberately visiting blue space, primarily on mental health and the promotion of physical activity. However, the authors highlighted that better methodological approaches, sampling strategies (randomized controls) and documented procedures, including evaluations are required to advance our knowledge on the topic (Gascon *et al.*, 2017). To our knowledge, no systematic review has been carried out that examines the benefits of therapeutic interventions in blue space.

The recognition of the importance of the value of nature and place as a determinant of wellbeing presents an opportunity to struggling healthcare systems seeking new and cost-effective services (Bragg and Atkins, 2016). The recent and rapid proliferation of NBS and interventions, such as the ‘green gym’ (Yerrell, 2008) and ‘blue gym’ initiative (Depledge and Bird, 2009) in the UK, is out-pacing policy and knowledge base. This creates challenges to understanding and assessing their impact for public health benefit (Raymond *et al.*, 2017). Better understanding of potential approaches and pathways are needed to gain an evidenced-based knowledge of the benefits of blue care.

### Aims of the systematic review

This evidence review aims to address the gap in understanding the health benefits of blue space within existing interventions for targeted individuals. It systematically identifies, summarizes and synthesizes studies that have examined the benefits, if any, of blue care for attaining or restoring psychological and/or physical health and wellbeing. This review examines the design, structure, benefits and outcomes as well as the mechanisms of intervention provision. Much of the literature on blue space is highly heterogeneous, varied in disciplinary origin, with authors approaching the study design using different methods and conceptualizations of blue space for health and wellbeing (or none at all) (Gascon *et al.*, 2017). As this is a recently emerging body of work and the available evidence remains highly heterogeneous, a narrative synthesis approach is used, which is textual rather than statistical (Lovell *et al.*, 2014).

This systematic review assesses existing peer-reviewed journal articles to identify and evaluate:

- Types and characteristics of BSIs—including use of validated methods and measures.
- Range of mechanisms, barriers or enablers associated with access to blue care.
- Range of the health and wellbeing outcomes measured.

Furthermore, the aim of this systematic review is to help provide evidence that can inform researchers, policy-makers and practitioners in the design and delivery of blue care for health promotion and restoration.

## METHODOLOGICAL APPROACH

### Search strategy

**Fig. 1:**
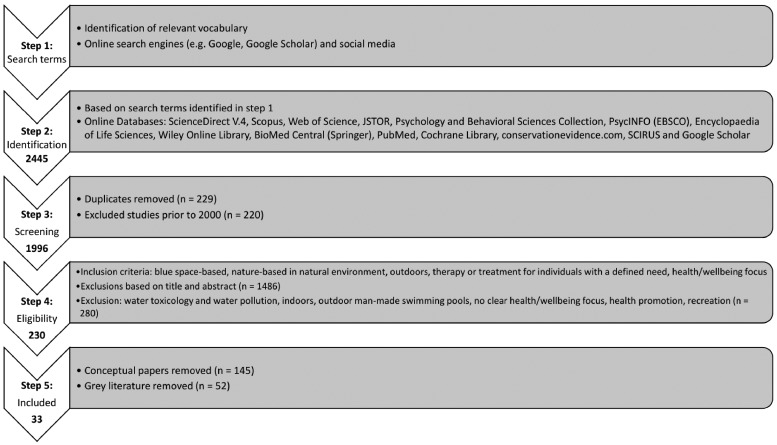
Summarized overview of the literature search and selection process.

From the initial search (Step 1 in Figure 1), several terms were identified (outlined below). The search included keywords, topic, title, abstract words. Literature searches included simultaneous computerized searches of online databases (Step 2, Figure 1). In addition, in a process of chain-referral sampling, authors’ publications, articles citing papers and reference list checking were carried out to obtain access to more material. Based on recommendations by Hartig *et al.* (Hartig *et al.*, 2014) a combination of nature (including blue space) terms, health and wellbeing terms, interactions, interventions and outcomes, sample, study type, behaviours, etc. were used (Supplementary Appendix S1).

### Exclusion/inclusion criteria

Two of the review authors (Carlin and Britton) co-developed inclusion and exclusion criteria and were verified by another review author (Kindermann) to see if the criteria were clear and applicable. Inclusion and exclusion criteria set out in Steps 3 and 4 (Figure 1) were applied. Searches were restricted to articles in the English language. Study populations in both urban and rural spaces were permitted and an unlimited, global geographic scope, including all target populations was applied. Only studies with a nature-based therapy or treatment intervention specifically targeted, designed or structured for individuals or a voluntary group were included. The study did not have to explicitly define ‘blue/green’ spaces. However, studies had to address outdoor, natural (i.e. non-manmade), blue space (e.g. rivers, lakes, coasts, sea, etc.) in relation to health and/or wellbeing (including studies where improving individual psychological, social and/or physical wellbeing was the primary goal of the intervention).

### Data extraction and collation

The research team adapted and co-developed a checklist of 60 questions (see Supplementary Appendix S2) from a previous desk-based study by one of the authors (Carlin *et al.*, 2017). The Cochrane, Campbell and PRISMA guidelines were used to ensure a systematic and consistent approach was applied by all three researchers in retrieving information to assess quality and decide on inclusion/exclusion from review (Cochrane, 2017). This included the recording and evaluation of details. Data were recorded using a structured template and ENDNOTE X7 was used to manage bibliographical information.

### Data analysis

The authors reviewed the data extracted independently to ensure all researchers were extracting the same information. The data were reviewed in the light of verification feedback. A narrative synthesis approach was adopted by the authors and included a combination of vote-counting methods followed by thematic analysis. While the Cochrane Review guidelines set out a need to assess risk of bias in all included studies (Cochrane, 2017) this review deliberately did not assess the risk of bias or ‘internal validity’ in the intervention studies. The authors recognize that there is a risk of bias with all included interventions, which were all non-randomized studies and potential biases, including in particular selection bias (the samples were self-selected) and reporting bias, are likely to be greater for non-randomized studies compared with randomized controlled trials (RCTs) (Cochrane, 2017). A quality appraisal tool was not applied for similar reasons. However, RCTs pose a challenge in the context of public health as they are often difficult to apply in ‘real world situations’ [(Rütten *et al.*, 2019), p. 4], an issue the authors return to in the discussion.

## RESULTS

### Study characteristics

Following the selection criteria of Steps 4 and 5 (Figure 1), 33 studies were included in the final selection for this systematic review. These were published from 2004 to 2017, with the majority of studies published in the last 5 years (Table 1).

**Table 1:** Study design characteristics

Author(s), year, country	Aims of Study	Sample size	Age/gender of participants	Health characteristics of participants	Health outcome measured	Validated tools/measures	Study design methods
Rogers *et al.*, 2014, USA	Assess ocean therapy for war veterans seeking treatment for PTSD.	11	18+ (majority 24–30 years), M, F (*n*=3)	PTSD, depression	Mental health, PTSD	None	Pre–post, uncontrolled study,
Berger and Tiry, 2012, Israel	Explore experiential approach to nature therapy for mental health issues.	NS	NS, Adults	Emotional and psychiatric difficulties (include depression)	Mental health	None	Experimental, Qual.
Godfrey *et al.*, 2015, UK	Evaluate wellbeing outcomes of surfing intervention for youth.	136	8–18, youth (plus parents and referrers), M, F	Social exclusion, mental health issues; sensory issues.	Psycho-social wellbeing, behavioural	Stirling Children's Wellbeing Scale, (SCWBS)	Pre–post, Quant., questionnaire
Caddick *et al.*, 2015, UK	Investigate wellbeing outcomes of surfing for combat veterans experiencing PTSD.	16	27–60, adult, M	PTSD	Wellbeing, PTSD	None	Qual., interview, PO
Clapham *et al.*, 2014, USA	Understand health benefits of surf programme for youth with disabilities.	17	5–17, youth, M, F	Mixed disabilities (physical, cognitive, behavioural)	Physical, psychosocial	Brockport Physical Fitness Test (BPFT)	Pre–post, Quant
Dustin *et al.*, 2011, USA	Explore therapeutic benefits of river running for veterans with PTSD	13	NS, Adults, M, F (*n*=3)	PTSD	PTSD	None	Qual.
Tardona, 2011, USA	Investigate wellbeing impacts of kayaking as a nature-based activity for inner-city youth	129	9–17, youth, M, F (17%)	NS	Wellbeing, behavioural	None	Qual., NS
Carin-Levy and Jones, 2007, UK	Investigate psychosocial benefits of scuba diving for individuals with acquired physical impairments	3	33–53, adult, M	Amputees and SCI	QoL, psychosocial, physical impairments	None	Post, Qual., interviews
Casey *et al.*, 2009, Ireland	Understand meaning and value of engaging in kayaking as a leisure pursuit for adults with a SCI.	6	NS, adults, M. F	SCI: quadriplegic, paraplegic	SCI	None	Post, Qual., interviews
White *et al.*, 2016, UK	Evaluate effects of sail training for adults recovering from drug and alcohol addiction	11	26–61, adult, M, F (*n*=3)	Addiction (drugs and alcohol)	Recovery from drug and alcohol addiction	None	Pre–post, Qual, interviews
Mowatt and Bennett, 2011, USA	Impact of therapeutic fly-fishing intervention on PTSD symptoms of war veterans	67	NS, adult	PTSD	PTSD	None	Qual., self-reflective letters
Nielsen and Mitchell, 2002, Canada	Investigate psychosocial impact of dragon boat racing (DBR) as post cancer rehabilitation both physically and emotionally.	6	43–75, adult, F	Breast cancer	Psycho-social	None	Qual. Interviews
McDonough *et al.*, 2008, Canada	Explore changes in body image and social support experienced by breast cancer survivors during a novice season of DBR	14	46–60, adult, F	Breast cancer	Body image, social support, breast cancer	None	Pre–post, Qual, interviews
Parry, 2008, Canada	Understand contribution DBR makes to women's health and breast cancer survivorship	11	40–60s, adult, F	Breast cancer	Women’s health, breast cancer	None	Qual., interviews
Sabiston *et al.*, 2007, Canada	Explore breast cancer survivors experiences of participation, motivation, social support, and physical self-perception related to their participation in DBR	20	42–70, adult, F	Breast cancer	Social support, physical self-perception, breast cancer	None	NS
Mitchell *et al.*, 2007, Canada	Investigate the psychosocial impact of DBR participation on women treated for breast cancer.	10	35–70, adult, F	Breast cancer	Psycho-social, breast cancer	None	Pre–post, mixed, questionnaire, interviews
Parry, 2007, Canada	Investigate broader health benefits of participation in DBR	12	40–60s, adult, F	Breast cancer	Broader health, breast cancer	None	NS
Unruh and Elvin, 2004, Canada	Explore impact of DBR on psychological wellbeing from point of view of breast cancer survivors.	3	50s, adult, F	Breast cancer	Psych. wellbeing, breast cancer	None	NS
Armitano *et al.*, 2015, USA	Explore benefits of surfing for youth with disabilities by assessing for physiological improvements.	16	5–18, youth, M, F	Cognitive and learning disabilities [Down Syndrome, Autism Spectrum Disorders (ASD), Microcephaly, Global Developmental Delays, Dandy-Walker syndrome], heart defects, hypothyroidism	Physiological, disabilities	BPFT	Pre–post, Quant,
Cavanaugh and Rademacher, 2014, USA	Determine benefits and outcomes of 2-day surf camp on social competence, social skills and self-concept of students with ASD	11	10–16, youth, M, F (*n*=3)	ASD	Psycho-social, social skills, self-concept, ASD	Social Skills Improvement System; Piers-Harris Children’s Self-concept Scale; Parent Perceptions of surf camp curriculum; SURF Skills Observation Checklist	Pre–post, mixed, multiple measures/scales
Colpus and Taylor, 2014, UK	Show the positive effects surfing has on young people with varying mental health conditions, personal and social needs.	72	8–17, youth, M, F	Mental health issues, social/personal developmental issues	Mental health, social needs	Wellbeing scale (0–10) / 6 measures	Pre–post, mixed, scale, focus groups
Lopes, 2015, Portugal	Demonstrate how surfing can be used to promote physical and mental health, social interaction and inclusion of persons with disabilities, regardless of age or disability.	321	8–66, all ages, M, F	Physical (SCI, amputees) and cognitive disability, visually impaired	Physical and mental wellbeing, social interaction, disabilities	None	NS
Hignett *et al.*, 2017, UK	Evaluate the impact of a surfing programme aimed at at-risk youth.	58	13–16, youth, M, F (*n*=10)	At-risk youth, behavioural issues, learning difficulties. Physically aggressive behaviours	Physiological, anti-social behaviour, wellbeing, connectedness	Physiological indicators (HR, SBP, DBP), self-reported wellbeing (BHPS-Y); Connectedness (adapted Inclusion of Nature in the Self-INS Scale); teacher ratings of Social and Emotional Aspects of Learning (SEAL) questionnaire.	Pre–post, mixed, interviews, questionnaire
Capurso and Borsci, 2013, Italy	Investigate the impact of a sail training on self-concept of adolescents.	147	13.18 (mean age), youth, M, F	Chronic disease or physical or cognitive disability.	Self-concept	MCSC scale	Quasi-experimental, pre-during-post (3 months), Quant, scale
McCulloch *et al.*, 2010, multi-country	Investigate purposes, beliefs and benefits of participation in sail training for youth, especially the social nature of the experience.	325	14–21, young adult, M, F	NS	Social impacts, social confidence	Self-assessment scale for social confidence	Pre–post (3 months), Mixed, interviews, scale
Bennett *et al.*, 2014, USA	Understand participants' perceptions of programme for veterans with combat-related disabilities.	28	22–50, adult, M, F (*n*=8)	PTSD, traumatic brain Injury, hearing and visual impairments	Combat-related disabilities	None	Qual., focus groups
Vella *et al.*, 2013, USA	Evaluate effectiveness of a fly-fishing programme in reducing the psychological concomitants of stress among a sample of veterans with PTSD.	74	22–64, adult, M, F (*n*=5)	PTSD, major depressive disorder, traumatic brain injury	Psychological (mood, stress, sleep), PTSD	PCL-M—degree of PTSD symptoms; Brief Symptom Inventory (BSI); PANAS—mood; PSS - stress; Pittsburgh Sleep Quality Index	Pre-during-post (6 weeks), Mixed
Ritchie *et al.*, 2015, Canada	Examine qualitatively how OALE promoted resilience and wellbeing for First Nations youth from one community population.	43	12–18, adolescents, M, F (*n*=5)	NS	Resilience and other aspects of health and wellbeing	None	Qual. journals, interviews, focus groups. During-post (3 months).
Ritchie *et al.* 2014, Canada	Evaluate the impact of an OALE on the resilience and wellbeing of First Nations adolescents from one reserve community.	73	12–18, adolescents, M, F (*n*=16)	NS	Resilience and other aspects of health and wellbeing	Resilience (RS-14); Mental Component Score; Physical Component Score; Self-esteem Scale; Flourishing Scale; Scale of Positive and Negative Emotion (SPANE); Satisfaction with Life (SWL)	Mixed. pre–post (1 month, 1 year), comparison group, questionnaire
Hayhurst *et al.*, 2015, NZ	Examine potential for resilience to be enhanced in a group of youth participating in a developmental voyage.	272	16.55 (mean age), adolescents, M, F (*n*=72)	NS	Resilience, self-efficacy, social effectiveness, belonging	Resilience Scale (RS-15); Self-Description Questionnaire III; Self-efficacy and Social effectiveness scales; Sheldon and Bettencourt’s 3-item inclusion scale; Weather rating.	Mixed model design 2×2 (time of resilience assessment: use of control group); interval, pre–post
Grocott and Hunter, 2009, NZ	Assess global and domain specific self-esteem following a 10-day developmental voyage (short-term and long-term effect).	193	15–18, adolescents, M, F (*n*=119)	NS	Self-esteem	Self-Description Questionnaire (SDQ III)	Mixed model design (sex×self-esteem×time); interval, pre–post (3 months)
Matos *et al.* 2017, Portugal	Assess if behavioural problems decreased in at-risk youth and learning enhanced in self-regulation, social and emotional skills and belonging through surfing.	48	10–16, adolescents, M, F	NS	Psycho-social wellbeing	Strength and Difficulties Questionnaire (SDQ); Youth experiences survey	Quant. Pre–post
Fleischmann *et al.*, 2011, USA	Assess surfing as a multimodal treatment for patients with polytrauma.	1	21, adult, M	Amputee, burn injury brain injury, mild depression, opiate use for severe pain	Physical (mobility, balance, pain) and psychological.	NS (measure of opioid use over time)	Mixed, case study approach

M, male; F, female; NS, not specified; PTSD, post-traumatic stress disorder; ASD, autism spectrum disorder; Qual., qualitative design; Quant., quantitative design; pre–post, pre-test–post-test design; OALE, outdoor adventure leadership experience.

#### Study aims

The aims of the studies (Table 1) can be categorized as (i) evaluating or assessing the effectiveness of a BSI for treating, reducing or alleviating symptoms of specific conditions [e.g. post-traumatic stress disorder (PTSD), addiction], and/or (ii) investigating or exploring the wellbeing (physical, mental, psychosocial) impacts and outcomes of a BSI, and/or (iii) studies that focused more on understanding the impact of the BSIs on participants perceptions, values and beliefs.

#### Study participants

**Fig. 2:**
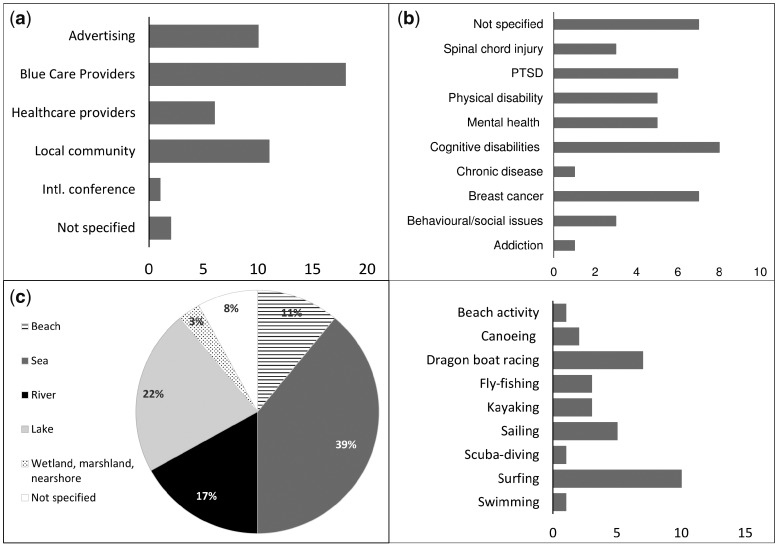
Results (numbers of studies) from analysis showing the recruitment procedures used in the studies (**a**), health characteristics of study population (**b**), type of blue space setting where interventions took place (**c**) and physical activities in blue care (**d**).

Taking all 33 studies combined, there were a total of 2031 participants. However, there were high levels of variation in sample size ranging from studies with as few as one participant (Fleischmann *et al.*, 2011) to over 300 (McCulloch *et al.*, 2010). Participants were primarily self-selected. The majority of studies recruited adults (*n* = 18), followed by youths (*n* = 12) (defined as pre-teen and teen, <18 years), one study recruited ‘young adults’ (14–21 years) and one study included a mix of all ages. Very few studies included participants aged over 65 years. Most studies included both male and female participants (*n* = 20), however, the majority in these mixed studies were predominantly male. Some notable exceptions included studies assessing sailing interventions in New Zealand, where the majority of the participants were female (Grocott and Hunter, 2009; Hayhurst *et al.*, 2015). Seven studies included women only, three were male-only, and two studies did not specify gender (Table 1). Participants were primarily recruited via organizers or practitioners delivering the intervention (*n* = 18), followed by local community networks (*n* = 11), advertising methods (*n* = 10) and healthcare providers (*n* = 6). In six studies participants were medically prescribed or referred by their healthcare provider (Figure 2a). Participants in other studies may also have been through professional medical referral procedures, however, this was not clearly stated.

The health characteristics of study populations varied (Figure 2b) with needs ranging from the physical to cognitive and psycho-social (Table 1). A large proportion of the studies included participants with multiple disorders or with a mix of health issues (*n* = 13). Mental health issues were the most prevalent (*n* = 17) yet the types of issues were very diverse and often overlapping. Specifically, these ranged from behavioural and social problems, typically among youth (*n* = 4), addiction (*n* = 1), depression or a major depressive disorder (*n* = 4), PTSD (*n* = 6), cognitive disabilities (mixed or unspecified) (*n* = 6), Autism Spectrum Disorder (ASD) (*n* = 2), traumatic brain injury (*n* = 3). Physical disease and disability included recovering breast cancer survivors (*n* = 7), heart defects (*n* = 1), spinal cord injury (*n* = 3), amputees (*n* = 3), visual and/or hearing impairment (*n* = 2), chronic disease (unspecified) (*n* = 1). Seven studies did not specify the health characteristics of the participants prior to the intervention.

#### Study measures and design

The primary aim of these studies was to explore and assess the various health and wellbeing outcomes and benefits of a particular BSI, mostly pilot interventions with small sample size. The primary health outcome assessed was mental health and/or psycho-social wellbeing, with one study measuring physical health exclusively (Armitano *et al.*, 2015). The majority of the studies were qualitative (*n* = 15), followed by a mixed-method design (*n* = 9) and five were quantitative (Table 1). A mix of methods and tools were used including semi-structured interviews (*n* = 13), questionnaires (*n* = 12) and other mixed methods (participant observation, field journals, focus groups, participant letters, practitioner reports), as well as validated and non-validated measures and scales (Table 1). Only 14 out of the 33 studies used validated measures to assess health and wellbeing outcomes. A varied mix of validated outcome measures were used to evaluate the effects of the intervention on mental health. Various physiological indicators and measures such as the Brockport Physical Fitness Test were used to assess physical health (Table 1). The majority of studies did not clearly report outcome measures or use any validated measures (*n* = 19). Most studies were used a pre-post design and only two used a comparison/control group (Ritchie *et al.*, 2014; Hayhurst *et al.*, 2015). The research process and intervention content were primarily pre-determined, rather than co-created with participants.

### General characteristics of interventions

Interventions ranged from a single day activity to 6 months for a single participant in a surf therapy intervention (Fleischmann *et al.*, 2011). Almost one-third of studies (*n* = 10) did not specify the duration of the interventions (Supplementary Appendix S3). These interventions were typically designed, led and facilitated by outdoor/adventure educators and providers, often within a charitable organization aimed at providing a type of ‘eco-therapy’ for specific groups. The main purpose of the interventions was health promotion, restoration and awareness (Table 1). There was little or no inclusion of participants in the design of these interventions. The few exceptions (*n* = 4) where the intervention was designed in response to the needs and aims of the group were Berger and Tiry (Berger and Tiry, 2012) for those with psychiatric disabilities, Ritchie *et al.* (Ritchie *et al.*, 2014, 2015) with Aboriginal adolescents in Canada, and Nielsen and Mitchell (Nielsen and Mitchell, 2002) with breast cancer survivors. Only seven studies listed funding sources for the interventions. These were primarily a foundation/charity or local authorities, and two were funded by the Big Lottery Fund (in the UK).

### Setting and type of activity

The majority of interventions took place in marine or coastal (*n* = 19), followed by freshwater (*n* = 14) environments; two included a mix of green and blue spaces (Berger and Tiry, 2012) and wetlands, marsh and nearshore (Tardona, 2011) (Figure 2c). Three studies did not define the type of outdoor blue space. Details about the blue space setting or the natural environment were limited in all studies and completely lacking from eight studies (Figure 2c). The interventions took place in a mix of both urban and rural environments. However, the majority of studies do not clearly situate the intervention in any setting (*n* = 12). Seven interventions took place in what authors describe as ‘wilderness’, seven in urban/semi-urban areas (e.g. city beaches, lakes) or a mixed urban/rural setting, and five took place out at sea. None of the studies provided a clear definition of blue space. The studies were primarily carried out in developed countries in Europe (*n* = 11), the USA (*n* = 10), Canada (*n* = 8), New Zealand (*n* = 2), one in Israel and one multi-country study (including Europe, USA, Australasia). There is greater emphasis on active experiences and physical activities rather than more passive activities in the interventions. The highest number of interventions were delivered through surfing (*n* = 11), followed by seven studies on Dragon Boat Racing (DBR), five on sailing, three fly-fishing and kayaking, two on canoeing, one located at the beach (as well as a forest park), swimming (as part of a kayaking intervention), and another on scuba diving (Figure 2d).

### Function and purpose of interventions

In over half (*n* = 17) of the studies, intervention aims and objectives were not clearly stated. Many of the programmes focused on the skills required to learn a new physical activity such as surfing or sailing with little modification or therapeutic addition. However, several studies emphasized a therapeutic approach such as ocean therapy based on principles of occupational therapy where, ‘participation in meaningful activities within the natural environment is both part of the therapeutic process and a desired outcome’ [(Rogers *et al.*, 2014), p. 397]. Berger and Tiry (Berger and Tiry, 2012) chose methods according to needs and aims of the group who were coping with emotional and psychiatric difficulties, highlighting the potential for creative processes to help adults better engage with nature as well as how nature can spark greater creativity. In Lopes (Lopes, 2015), the intervention was based on hydrotherapy but applied to a coastal environment rather than indoor pool setting. In Matos *et al.* (Matos *et al.*, 2017), ‘Surf-Salva Camp’ targeted ‘at-risk youth’ in Portugal and included psychologists and surf instructors in its design and delivery. The majority of the studies tended not to set specific targets but instead created a process whereby participants could experience respite from their symptoms. Caddick *et al.* [(Caddick *et al.*, 2015), p. 80] describe surfing as ‘a vehicle for pursuing pleasure and escaping pain rather than for loftier notions of psychological growth and development’, and Godfrey *et al.* [(Godfrey *et al.*, 2015), p. 26] state that surfing provides ‘a chance to forget rather than focus on problems’.

Very few studies (*n* = 4) assessed the effect of blue space activities on nature connectedness (in relation to the aims/objectives and measures used). For those that did, the intervention was designed in response to the characteristics of the local natural environment (Berger and Tiry, 2012). Caddick *et al.* (Caddick *et al.*, 2015) focused specifically on the effects that surf had on veterans’ wellbeing, in particular the sensory and embodied experiences veterans had while in the sea. Similarly, Lopes [(Lopes, 2015), p. 6] highlighted the influence of specific qualities of blue space in functional rehabilitation, including how, ‘the absence of gravity in saltwater improves mobility, which improves cardio-respiratory function and is an integral muscular workout’. Hignett *et al.* (Hignett *et al.*, 2017) included a specific measure of nature connectedness. However, it was not assessed in relation to a health or wellbeing outcome.

### Outcomes and benefits

Studies appraising mental health and psycho-social wellbeing outcomes (the most common health outcomes assessed) showed some improvement overall. The most commonly assessed wellbeing indicators included self-esteem, self-efficacy, social confidence, resilience and other psychological indicators (e.g. stress, mood) using self-report measures. Enhanced social relationships were also reported and pro-social behaviour (Supplementary Appendix S3). Improvement in environmental connectedness and the effect on health and wellbeing outcomes was less definitive. Environmental connectedness is linked with psychological restorativeness, although few large studies have explored what and how environmental qualities affect these outcomes (Wyles *et al.*, 2017b). Hignett *et al.* [(Hignett *et al.*, 2017), p. 12] found that ‘Surprisingly, there was no direct improvement in connectedness to nature or the beach as a result of the study overall’. Bennett *et al.* (Bennett *et al.*, 2014) referred to the importance of the physical setting for restoration, including the sound of the river but did not provide any details on the quality or characteristics of the natural setting. Hayhurst *et al.* (Hayhurst *et al.*, 2015) did not consider nature connectedness, but included a weather rating scale as part of a mix of measures assessing the effect of sail training on the resilience of young people.

Only six studies considered or assessed some aspect of physical health outcomes. Three surfing interventions with participants with mixed cognitive and physical disabilities measured physical health outcomes using the Brockport Physical Fitness test. However, the primary benefits reported in each study were mixed. Armitano *et al.* (Armitano *et al.*, 2015) reported improved numerous areas of physical fitness (upper-body strength, core strength, cardiorespiratory endurance). In Fleischmann *et al.* (Fleischmann *et al.*, 2011) specific features such as response to waves (movement) and skills required to surf (balance) were attributed to enhanced vestibular balance, as well as pain reduction and subsequent reduced dependency on narcotics attributed to the psychological effect of surfing. All seven studies on DBR found no negative physiological effects for participants recovering from breast cancer and some improvements in self-reported body image. There was a significant drop in heart rate among vulnerable youth after surfing, suggesting improved fitness (Hignett *et al.*, 2017).

The short-term benefits of the interventions were well reported. However, very few studies considered the long-term effects. Just over half of the studies used pre-post design, of these, only three assessed participant’s experiences during the intervention. Five studies considered longer-term effects, at 3 months (Grocott and Hunter, 2009; McCulloch *et al.*, 2010; Capurso and Borsci, 2013), 5 months (Hayhurst *et al.*, 2015) and 1 year after the intervention (Ritchie *et al.*, 2014), with contrasting results. Capurso and Borsci (Capurso and Borsci,2013) found that although self-concept (defined in the study as, ‘a multidimensional and context-dependent learned behavioural pattern that reflects an individual’s evaluations of past behaviours and experiences, influences an individual’s current behaviours, and predicts an individual’s future behaviours’, p. 16) increases after sailing it reverts back after 3 months. Hayhurst *et al.* (Hayhurst *et al.*, 2015) and Grocott and Hunter (Grocott and Hunter, 2009) reported that increased resilience and self-esteem were maintained 5 and 3 months, respectively, following sailing interventions. Ritchie *et al.* (Ritchie *et al.*, 2014), who used the same validated measure of resilience as Hayhurst *et al.* (Hayhurst *et al.*, 2015), found that resilience (defined in the study as, ‘the ability to successfully cope with change or misfortune’, p. 2526) reverted back to pre-intervention levels after 1 year.

### Barriers and adverse effects

The studies were evaluated to identify any barriers or adverse effects (Supplementary Appendix S4). Barriers are defined here to mean any factor that may inhibit or reduce a person’s ability to access or participate in BSIs. These barriers were identified and categorized by the review authors. Fourteen studies were identified as referring to some form of barrier in the description of the study. Barriers ranged from various access issues and lack of resources and equipment, to fears and stigma associated with personal abilities, level of fitness, environment, social and cultural norms and diagnosis of illness, and level and appropriateness of training for those delivering blue care. Thirteen studies did note some adverse effects. Adverse effects are any perceived negative effects experienced by those participating in the study during or after the intervention. These included feeling ‘emotionally low’ during or post-intervention as identified by participants suffering from PTSD in a surf therapy programme (Caddick *et al.*, 2015), by some survivors of breast cancer in DBR (Sabiston *et al.*, 2007; McDonough *et al.*, 2008; Parry, 2008) and post-sailing voyages (Capurso and Borsci, 2013; White *et al.*, 2017). Gender-based barriers (Tardona, 2011), seasickness and discomfort caused by poor weather conditions and tiredness or fatigue post-activity were also identified.

## DISCUSSION

This review systematically identified and synthesized studies that examined BSIs for promoting or restoring psychological and/or physical health and wellbeing. In the following section, the implications of the design, structure, function and outcomes as well as the mechanisms of intervention provision are discussed. Furthermore, the authors highlight limitations and gaps with recommendations for further study and research as well as implications for health promotion, policy and practice.

### Study and intervention design

The studies included in this review were highly heterogeneous, varied in disciplinary origin, with different approaches to study design and/or use of methods. They lacked conceptualizations of blue space and were limited in their use of validated measures. Social mixing in most of the interventions (i.e. participants with differing abilities or diagnoses, etc.) poses a challenge for research, especially where the emphasis is on clearly defined interventions and homogenous study populations within the medical literature (Sempik and Bragg, 2016). In the context of BSIs, the weakness of RCTs is that, ‘most RCTs focus on outcomes, not the process involved in implementing an intervention’ [(Oakley *et al.*, 2006), p. 413] and therefore fail to account for how social and environmental processes influence behaviour (Duane *et al.*, 2016). Furthermore, while RCTs would be desirable in this area of research, it is questionable how feasible implementing one would be considering: (i) blinding participants to an intervention arm especially when the activities they are undertaking are potentially coordinated by specialist organizations, (ii) the ethics of allocating participants to a control arm who may stand to benefit from the intervention arm and (iii) the financial resources often available to, e.g. surf schools or similar, who are likely to be delivering the intervention. Consequently, it may be sensible to expect and accept a lower standard of evidence from such intervention studies, at least at present.

Recruitment of participants lacked an even spread across socio-economic groups, age (elderly) and nationalities. In most cases contextual information regarding participants was not provided. Notably absent were studies from Latin America, Africa, Middle East and Asia. The review also highlights a lack of consideration of wider community, social support networks and services in intervention design and delivery. Poland *et al.* (Poland *et al.*, 2009) argue, that in addition to addressing the needs and capacities of people, health interventions need to address local contexts in order to assess the circumstances in which outcomes are achieved and the comparability of such findings. The duration of interventions (dosage) varied greatly. This review has identified that duration of an intervention is a knowledge gap in relation to sustained health outcomes. In Fleischmann *et al.* (Fleischmann *et al.*, 2011), a dramatic and sustained reduction in opioid use occurred after a 6-month surfing intervention. However, the majority are short-term or one-off pilot interventions (limited by funding) with little discussion of longer-term healthcare promotion and provision for participants. In typical medical trials, longer-term provision of interventions rely on robust evaluations in order to secure further funding and staff time. This in turn can be translated into applications for larger trials which explicitly outline mechanisms of health benefits. There is a need to consider how to build capacity after funds and expertise is withdrawn (Poland *et al.*, 2009). Furthermore, the ethical implications of this, although beyond the scope of this paper, deserve further investigation. As recommended in other reviews on health-based interventions (Lefebvre and Flora, 1988; Campbell *et al.*, 2000; Poland *et al.*, 2009) better means of evaluating the impact of nature-based programmes on public health are needed.

There was very weak involvement of participants’ perspectives in the design and delivery of interventions, and participants’ perceived experience of blue space was often lacking. Studies provided limited details regarding participants’ attitudes towards particular environments or how they might have previously engaged with nature. Evidence reviewing effective health-based interventions emphasizes the need for greater engagement with participants in the design and delivery as well as an understanding of how participant expectations and individual needs measure against actual outcomes (Poland *et al.*, 2009; Rütten *et al.*, 2019). Some studies in this review did engage in a more collaborative process that included health professionals, outdoor educators and researchers in the design. By working together, community members can gain a sense of ownership that will sustain their interest and commitment to the intervention and make it more likely that the intervention will be integrated into existing community structures (Bryant *et al.*, 2009). The increasing tendency to engage participants in both the conceptualization of interventions and the interpretation of their outputs is seen as a move towards validation based on reality (Domegan *et al.*, 2017; Rütten *et al.*, 2019). Studies show that co-created interventions can lead to more sustained outcomes and greater participation (Duane *et al.*, 2016). However, participants may not always know what intervention components may be successful at affecting outcomes; e.g. they may exhibit affective forecasting errors (Wilson and Gilbert, 2005). Only one study included a long-term follow-up (Ritchie *et al.* 2014) to assess the potential for sustained health and wellbeing benefits (i.e. >6 months), but a tendency to focus on short-term outcomes is typical of health-based interventions in general (Nutbeam, 1998; Campbell *et al.*, 2000). This could be due to a lack of funding for longer-term evaluations or the lack of theoretical explanations of behavioural maintenance as opposed to behavioural initiation (Kwasnicka *et al.*, 2016).

### Activity and setting

The interventions in these studies were not designed with the intention of conducting research nor were the activities developed for any purpose other than the treatment, therapy or recovery of participants. Greater collaboration between researchers, practitioners and community, as in Ritchie *et al.* (Ritchie *et al.*, 2015), could help build a more coherent evidence base and communicate effectiveness to policy-makers (see Rütten *et al.*, 2019). Further experimental and controlled interventions could be designed to help inform policy and practice [see, e.g. (Wyles *et al.*, 2017a,b)]. The importance and impact of the physical setting on health outcomes and determining what proportion of the health benefit is attributable to the natural environment as opposed to other factors was poorly considered in the studies. The types of activities used to deliver BSIs were typically classified in the action-sports sector or requiring learned skills, with a tendency to emphasize the immersive and experiential qualities of these activities in blue space. Notable absentees from the type of activities used in blue care include activities that are typically more accessible such as walking, running or even swimming (Foley, 2015). These are activities which require very little in the way of resources or indeed funding. Unlike green care interventions (Sempik and Bragg, 2016) passive and conservation-based activities and approaches are somewhat lacking in blue care. The emphasis on more physically challenging interventions that might act as a barrier for some, could explain a lack of inclusion of elderly participants. However, this also points to a larger issue of (mis)perceptions and stereotyping that persists in public policy, practice and research around ageing and the outdoors (Wheaton, 2017). The specialization of these interventions both in terms of activity type, volunteer/practitioner training requirements, equipment, suitable environments, target group, can, as Hignett *et al.* (Hignett *et al.*, 2017) commented, lead to an exclusionary attitude and belief that it’s ‘not really for us’. The ethical implication of this merits further study. That said, studies do exist on walking interventions in blue space [e.g. river paths (Marselle *et al.*, 2013)], however, these often do not have populations with a defined need participating.

The authors acknowledge that there is an extensive body of research on the topic of water-based or hydro/aquatic therapy, as well as cryo-therapy (e.g. cold-water immersion) and increasing uptake of ‘wild swimming’, especially for women (Smolander *et al.*, 2004; Thorsby, 2013). However, these experimental studies are typically carried out indoors, or in man-made settings where environmental factors can be controlled or are studied as recreational activities for general health promotion. This tension between controlled experiments and more complex, community interventions is highlighted elsewhere in the literature, with no single methodology being advocated (Nutbeam, 1998; Domegan *et al.*, 2016; Komro *et al.*, 2016; Wong *et al.*, 2017). Furthermore, swimming is usually more accessible in non-natural environments (e.g. swimming pools), which might explain why it does not appear as often as, e.g. surfing which can only really be practiced at the coast. A comparison of the various mechanisms with which people engage with blue space, in both complex and controlled interventions is an area for further study. Blue care design could benefit from a better understanding of how environmental preferences and characteristics, such as wildlife and perceived biodiversity, can enhance wellbeing (Wyles *et al.*, 2016, 2017b; Carlin *et al.*, 2017; Cracknell *et al.*, 2017; White *et al.*, 2017). In addition, why preference is given to some activities (e.g. surfing) over others (e.g. swimming or walking), needs further investigation. Studies by Ritchie *et al.* (Ritchie *et al.*, 2014, 2015) uniquely included a cultural component of nature connection and its relevance for the learning and change process that occurred in response to the intervention, as well as how this might intersect with other determinants of health such as gender, race and ethnicity. Further evidence is needed to comprehend the drivers and components of a successful BSI, such as the difference between settings and activities across interventions.

### Function and outcomes

Overall, positive outcomes were identified for health and wellbeing, especially mental health and psycho-social wellbeing in the short term. Some interpersonal as well as individual effects were evident with a number of studies placing strong emphasis on social connection, sense of belonging, and interaction with others who have shared life experiences, as well as the connective properties of water environments. The findings suggest how activities in blue space, rather than particular qualities of blue space, might contribute to rehabilitation and health promotion (Lopes, 2015; Fleischmann *et al.*, 2011). Water can be particularly therapeutic, altering bodily sensations and levelling the playing field, e.g. with participants feeling equal to non-disabled divers (Carin-Levy and Jones, 2007). The number of studies assessing the physical impacts of blue care were very limited in comparison to mental health, unsurprising given that the majority of populations included in the studies were characterized with mental rather than physical health issues. However, this raises the question of why these population groups are not more targeted for these interventions. Perhaps another reason might be the difficulty in designing controlled or clinical interventions in an outdoor, natural water setting with physical tests measured more effectively when designed as experiments in controlled environments, typically indoors (Smolander *et al*., 2004; Collier *et al.*, 2015). The findings emphasize a multi-dimensional view of health with participants experiencing positive changes to sense of self, health and wellbeing, as illustrated in the following quote from a participant in a scuba diving intervention [(Carin-Levy and Jones, 2007), p. 10], ‘Diving turns me back into a human being, I go down there and I’ve got the freedom and I’m back to being a person’. However, not all experiences were positive. Participant selection bias could favour those who had more positive experiences, especially in qualitative studies where a small number of participants from a large sample might only be interviewed [e.g. White *et al.* (White *et al.*, 2016) only interviewed 11 out of the 100 participants on sail training trips].

A number of studies (*n* = 9) did not clearly specify the aims of the interventions they were assessing. Interventions designed without clear aims or objectives hinder the ability to understand or evaluate the impact. Although beyond the scope of this paper, the number and range of barriers and adverse effects highlights the complexity of blue care design and delivery. Challenge can be an important factor for enjoyment and quality of life enhancement. For example, self-reported feelings of tiredness, cold, body aches were also considered factors that led to a sense of self-efficacy and perseverance (Ritchie *et al.*, 2015), and challenging activities were linked to greater sustainable wellbeing (Hignett *et al.*, 2017). However, the ‘mood-dip’ identified by some studies can be caused by perceived discrepancies between personal experience during an intervention and the social requirements or demands of daily life after an intervention (Capurso and Borsci, 2013). This highlights the need to better understand the barriers to engaging with blue space for wellbeing (Pitt, 2018). There is also a need for a contextually sensitive and process-oriented approach with process evaluations—measuring more than ‘what’ worked well; but also evaluating ‘how’ and ‘why’ success or indeed failure happened (Oakley *et al.*, 2006; Rütten *et al.*, 2019; McHugh *et al.*, 2018). Further research is needed on how unintended consequences might be identified and the longer-term impact of BSIs.

A key consideration for public health promotion is how participants are referred or gain access to interventions. For example, in this review, to qualify for a BSI those suffering from PTSD required a clinical diagnosis. This poses a barrier for a mental health issue that is stigmatized and often goes unreported or untreated. Furthermore, studies lacked a thorough description of practitioner roles, levels of expertise and skills used in the intervention process. This perhaps highlights the need for training to facilitate nature encounters for health and wellbeing across sectors in outdoor public spaces (Maller *et al.*, 2006).

### Limitations of the studies

The review process identified some of the following limitations of the studies. Due to the small sample size of nearly all the studies as well as self-selection bias, lack of control groups or long-term follow-up, the risk of bias was moderate to high for all studies and limits the transferability of the findings. To some extent, as most of this research has only emerged in the last decade, this is to be expected. There was a notable lack of diversity in participant selection and/or poor description of participant characteristics in some studies, with the majority of participants being Caucasian, well-educated and from mid to high income backgrounds. Another common limitation was the risk of gender bias. Additionally, poor consideration was given to the potential gendered effects of interventions, e.g. the increased likelihood of female participants dropping out of the surfing intervention programme as noted by Godfrey *et al.* (Godfrey *et al.*, 2015). A lack of validated measures might suggest a lack of available tools for assessing health/wellbeing outcomes. However, it is more likely that there are too many to choose from and that there is instead a lack of measures specifically designed to meet the particular needs of a target population and place (Linton *et al.*, 2016). Given that BSIs offer an alternative to more medicalized interventions some participants may feel uncomfortable with being evaluated by measures that are overly focused on the health issue or ‘problem’.

Despite the importance of understanding connectedness to nature as a prerequisite for health outcomes (Schultz, 2002), it was given very little attention within the studies. A consistent lack of description of setting characteristics or the natural environment as a ‘subject’ was evident across all of the studies despite nature/water being mentioned frequently by participants as beneficial for their overall sense of wellbeing. Some exceptions were Tardona (Tardona, 2011), who made reference to participants’ appreciation of the natural environment and the calming effect of water as well as noting biodiversity and environmental characteristics, and the influence of climatic/weather conditions (both positive and negative). The how and why a particular nature setting was selected would strengthen the interpretation of intervention outcomes (Poland *et al.*, 2009). 

The conceptual ambiguity of terms such as ‘blue space’, ‘health’ and ‘wellbeing’ invites both narrow and vast definitions and exacerbates a lack of coherence and common language for nature-based providers, researchers and policy-makers, an issue highlighted in a report by Natural England (Bragg and Atkins, 2016). Ambiguity around the use of ‘wellbeing’ and associated terms (e.g. self-esteem, resilience) in a place-based context persists. BSIs would benefit from a common language to describe subjective wellbeing across nature and health research, policy and practice. There is an historic and recent precedent for the inclusion of common set of cognitive and experiential components of subjective wellbeing (Linton *et al.*, 2016). This would allow comparability and harmonization of findings, and as a consequence have greater relevance for policymakers. However, as identified in a recent review of wellbeing in the UK, wellbeing measures are often highly individualized and fail to account for the socio-ecological factors of disadvantage and social inequality (Mansfield *et al.*, 2018). Furthermore, as highlighted by Ritchie *et al.* (Ritchie *et al.*, 2015), dominant, individualized measures of wellbeing fail to account for indigenous models of wellbeing embedded in a socio-ecological context. 

Studies could also benefit from drawing on a more inter-disciplinary framework such as a complex systems approach taking a non-linear perspective (Savigny and Adam, 2009). Briefly, a ‘system’ is a set of elements—e.g., people, organizations, etc.—interconnected in such a way that they produce their own pattern of behaviour over time (Meadows, 2008; Domegan *et al.*, 2017). It assumes multi-causality at work between the diversity of blue-green forces and health with dynamic interactions and feedback muddying the waters. In contrast, linear interventions within the epistemology of classical science are not sensitive enough to the dynamics and complexities of nature-based messy or ill-structured problems. In this non-linear setting, stakeholders and their engagement are central to success of messy or ill-structured problems (Jonassen, 2003). The boundaries of blue space and nature-based issues are diffuse (Hisschemöller and Gupta, 1999). Outcomes are best seen as an interactive process with a multitude of stakeholders who are interrelated, not independent (Bryson, 2004). This translates into a system of stakeholders; a dispersed spectrum of individuals and groups with common interests, extending beyond a traditional participant intervention focus. That said, with this complexity, there is the risk that it becomes more difficult to develop process evaluations which accurately evaluate the contributions of the setting, activity, role of participant and researcher.

## CONCLUSION

This is the first systematic review of the literature on therapeutic BSIs and it shows that interventions are diverse in study population, setting and activity. The majority of studies included adults (although not elderly) often with multiple disorders, predominantly psychological. The studies were primarily in developed countries and the emphasis was on active (rather than passive) activities with marine or coastal settings favoured. Findings suggest how activities in blue space, rather than particular qualities of blue space, might contribute to rehabilitation and health promotion. Many of the interventions resulted in significant positive effects for health, especially psycho-social wellbeing benefits, with relatively few findings for physical health. This review illustrates that blue care has the potential to improve mental health for diverse groups, but more research is required, and we call for further investigations into BSIs. In particular, more rigorous pilot interventions co-designed in collaboration with population groups, professionals, policymakers and researchers are needed to evaluate outcomes.

With a lack of longitudinal studies, it remains untested whether the benefits associated with participation in blue space are sustained, as well as how this relationship to blue space could vary across the lifecourse (Pearce *et al.*, 2016). The evidence is highly heterogeneous in study design, method and measurement with high risk of bias making it difficult to determine the impact of blue care on health and wellbeing. The design and delivery of BSI’s would benefit from a more detailed evaluation of outcomes. Studies would benefit from both broad and in-depth understanding of the association and evidence between blue space and health outcomes. We advocate a complex systems approach that considers the complexity of multiple stakeholder groups and how they simultaneously affect and are affected by an intervention. As discussed, a contextually sensitive approach that considers participation, process evaluations and dynamic understandings with multiple stakeholders is needed. There is a tendency to count only the ‘good interactions’, however, this review also highlighted potential for negative experiences and a need to unpack potential risks and trade-offs for vulnerable groups. The rapid proliferation of nature-based interventions threatens to out-pace the knowledge base of meaningful and appropriate strategies for public health benefit. This review highlights the need to improve our understanding of complex nature-based interventions for health outcomes. Investment in further research is needed to understand the general significance of blue space for public health and the potential for embedding blue care within existing health promotion services.

## FUNDING

This study is part of the NEAR Health project, funded by the Environmental Protection Agency (EPA) and the Health Service Executive under Grant Award No. 2015-HW-MS-2.

## Supplementary Material

day103_Supplementary_Data
