# Supplementary material for: Blue care: a systematic review of blue space interventions for health and wellbeing
Source: Health Promot Int. 2018 Dec 18;35(1):50–69. doi: 10.1093/heapro/day103 (PMC7245048; doi:10.1093/heapro/day103)
Supplement: day103_Supplementary_Data [file day103_supplementary_data.zip › day103-Suppl_data/Supplementary_Appendix 2.pdf]

NEAR health lit review

Publication details: Systematic review of blue and green space for health and wellbeing literature. Key search words: green/blue spaces, water, health, coastal, marine, wellbeing, nature/outdoor/adventure/aquatic/balneo therapy, stakeholder/ evidence/methodology TBA

Q1: Author(s)

Q2: Year

Q3: Title of publication

Q4: Select Publication type: Journal/Book/Conference Abstract/ Conference proceedings (published)

Q5: Name of Publication / Journal

Q6: Journal Discipline: tick all that apply

multidisciplinary/ Geography /Landscape /Tourism /Sport (active) /Health /Science /Psychology /Recreation /Environment /Education /Therapy /Medicine /Spatial Planning /Land Use /Conservation /Ecological Economics /Social Marketing /Social Innovation /Ecology / Wellbeing /Arts

Q7: Link to citation:

Q8: Publication Keywords (if any) (NS - not specified)

Q9: Country(ies) of Origin (where study took place); if review, state

Q10: Source of funding (if any)

NEAR Health lit review: Study characteristics: aims, objectives, theme, design, participants, setting etc.

Q11: Study type – choose from Qualitative/Quantitative/Mixed/Other

Q12: Study type (tick all that apply)

- systematic review
- review
- survey
- questionnaire
- mixed methods
- fieldwork
- experimental
- focus groups
- semi structured interview
- structured interview
- panel
- purely theoretical
- Other:

Q13: Study question - what health/wellbeing question is being addressed

Q14: What is the study aim?

Q15: What are the study objectives

Q16: Did the research identify any theme(s)? See discussion for breakdown of topics or commonalities with other research (point is to spot emerging trends/themes within research/practice community)

Q17: Give details of the study design, incl. methods used.

Q18: List tools used, if any

Q19: List blue/green space indicators used, if any

Q20: Study inclusion and exclusion criteria, if any

Q21: Recruitment procedures used (e.g. details of randomisation, blinding)

Q22: Unit of allocation (participant, GP practice, community etc) (This is to do with sampling, appropriateness and bias)

Q23: Number of participants

Q24: Characteristics of participants at the beginning of the study e.g. conditions outlined etc

Q25: Age cohort (tick all that apply)

- 0-11
- 12-19
- 20-29
- 30-69
- 70+
- not specified
- not applicable
- Other:

Q26 Gender (tick all that apply)

- Female
- Male

- Both
- Not specified
- Not applicable
- Other

Q27: Ethnicity

Q28: Socio-economic status

Q29: List any comorbidities noted (simultaneous coexistence of multiple disorders alongside a primary disorder)

Q30: Disease/condition characteristics

Q31: Setting in which activity/intervention took place

Q32: Description of activity/intervention

Q33: List barriers identified, if any

Q34: Reference to space types (tick all that apply)

- Blue
- Green
- Both
- Other (e.g. grey/brown etc)
- Not specified
- Nature encounters outside of specific green/blue spaces

Q35: Are blue / green spaces defined?

- Yes
- No

Q36: Provide definition of blue/green space given in the study, if applicable

Q37a: Ecosystem spaces in study (tick all that apply)

- Marine
- Coastal
- Freshwater
- Wetland
- Grassland

- Forest
- Desert
- Montane
- Not applicable
- Other:

Q37b: "Created" ecosystems listed in study

- Zoo
- Aquarium
- Wildlife Park
- Petting Farm
- Not applicable
- Not specified
- Other:

Q38: Study location (tick all that apply)

- Rural
- Urban
- Mixed
- "Wilderness"
- Not applicable
- Other:

Q39: List space use (s) in study

- Amenity (playground)
- Amenity (sports pitch)
- Park (has habitats/ecosystems such as woods/meadows and not just ornamental borders or amenity areas)
- Outdoor Gym
- National Park
- Beach
- Foreshore
- Ocean
- Not applicable
- Other:

Q40: List any site designations, if any

- Natura network (SPA, SAC)
- World Heritage Site
- GeoPark
- Nationally protected site (AONB/NHA/)

- RAMSAR
- Marine Protected Area
- IUCN protected area
- Not applicable
- Not specified
- Other:

NEAR Health Lit. Review - Study Outcome data/results

Q41: Unit of assessment/analysis?

Q42: Statistical techniques, if any

Q43: Outcomes of study (wrt aims/objectives)

Q44: Any adverse events noted?

- Yes
- No

Q45: Give details of adverse events, if any

Q46: Did authors note any additional outcomes (unintended)

- Yes
- No

Q47: List details of intended additional outcomes noted, if any

Q48: Study recommendations

Q49: Research impacts and implications

Q50: Give details of costs indicated in study, if any

Q51: Give details of resource requirements indicated in study, if any

Reviewer Synopsis

Q52: Outline brief synopsis here

Q53: Any overall comment?

Q54: Links or important points for other researcher?

Q55: Link to/corresponds with other papers (if aware of connection)

Q56: Appropriateness of methods?

Q57: Bias in study

Q58: Gaps Identified

- By authors
- by researcher

Q59: List gaps highlighted in paper

Q60: List gaps highlighted by researcher

*Reviewer name:
