# Supplementary material for: Blue care: a systematic review of blue space interventions for health and wellbeing
Source: Health Promot Int. 2018 Dec 18;35(1):50–69. doi: 10.1093/heapro/day103 (PMC7245048; doi:10.1093/heapro/day103)
Supplement: day103_Supplementary_Data [file day103_supplementary_data.zip › day103-Suppl_data/Supplementary_Appendix 3.docx]

**Appendix 3.** Main characteristics of blue care design

| Author (s), year, country | Duration | Blue space setting | Type of intervention activity | Main outcomes |
| --- | --- | --- | --- | --- |
| Rogers et al.., 2014, USA | 5 wks (1 x wk) | Beach, sea | Surfing | Reduced PTSD and depressive symptom severity post-intervention. |
| Berger and Tiry., 2012, Israel | 1 day | Forest park; beach | Nature therapy | Potential benefits of outdoor art therapy - in nature for adults coping with mental health difficulties. |
| Godfrey et al.,2015, UK | 6 wks (1 x wk) | Sea | Surfing | Significant and sustained increase in wellbeing from surfing activity |
| Caddick et al., 2015, UK | 21wks (2 x wk, 3 wk residential) | Sea | Surfing | Sense of respite from PTSD; Emotional benefits limited to time spent in water. |
| Clapham et al., 2014, USA | 8 wk (2 x wk) | Sea | Surfing | Gains in social development and self-confidence |
| Dustin et al.,2011, USA | 4 days | River | Kayaking | Reduced PTSD symptom severity, enhanced perceived coping skills, confidence and self-efficacy. |
| Tardona., 2011, USA | 1 day (6hrs) | Wetland, marsh, nearshore | Kayaking, swimming | Positive wellbeing effects reported, including appreciation for natural environment. |
| Carin-Levy and Jones, 2007, UK | NS | NS, Sub-aqua | scuba diving | Social connection, improved sense of self-worth, physical respite (body sensation of weightlessness), feeling free from impairment. |
| Casey et al., 2009., Ireland | NS | NS, sea | kayaking | Sense of freedom and equality facilitated by freedom of mobility on the water |
| White et al., 2016, UK | 5 days | Sea | sailing | New competencies developed and greater self-confidence reported. |
| Mowatt and Bennett, 2011, USA | 2 days | River | fly-fishing | Restorative benefits of fly-fishing identified |
| Nielsen and Mitchell, 2002, Canada | year round training | NS | dragon boat racing (DBR) | Increased sense of wellbeing and social support. |
| McDonough, et al., 2008, Canada | NS | Lake / outdoor still-water body | DBR | Positive psychosocial QoL changes. Shift from a focus on physical appearance to physical functioning. Social connection. |
| Parry, 2008, Canada | NS | NS, Lake? | DBR | Contribution to social, emotional, physical, spiritual, and mental health. |
| Sabiston et al., 2007, Canada | NS | NS, Lake? | DBR | Psychosocial benefit, social support, overcome physical challenges; Varied experiences of psychological growth. |
| Mitchell et al., 2007, Canada | NS | NS, Lake? | DBR | Short involvement impacted QoL. Experience an uplifting sense of freedom, power and control. |
| Parry, 2007, Canada | NS | NS, Lake? | DBR | Changes to sense of self, personal identity, and health and well-being. Although not all experiences positive. |
| Unruh and Elvin, 2004, Canada | 8 wks  (1 x wk) | NS, Lake? | DBR | Benefits physical and emotional wellbeing, social support; decreased stress |
| Armitano et al., 2015, USA | NS | Sea | Surfing | Improved physical fitness - upper-body strength, core strength, as well as cardiorespiratory endurance. |
| Cavanaugh and Rademacher, 2014, USA | 6 wks  (1 x wk) | Sea | Surfing | Improved social competence, social skills and self-concept (No statistical significance in overall outcome scores) |
| Colpus and Taylor, 2014, UK | 1 day | Sea | Surfing | Improvement of confidence, self-esteem and wellbeing, motor and social skills, behaviour and re-engagement with school. |
| Lopes, 2015, Portugal | 12 wks  (1 x wk) | Sea, beach | Surfing | Social interaction, multi-sensory stimulation, during and after pleasure, autonomy and happiness. |
| Hignett et al., 2017, UK | NS | Sea, beach | Surfing | Significant drops in heart-rate, increased satisfaction with appearance, more positive attitudes towards school and friendships, greater environmental awareness, and more positive teacher evaluations, post-intervention. No direct improvement in connectedness to nature or the beach |
| Capurso and Borsci, 2013, Italy | 5-15 days | Sea | sailing | Self-concept increases after but it reverts back after time (3 months). |
| McCulloch et al., 2010, Multi-country | 4 days | Sea | sailing | Social confidence and capacity to work collaboratively with others. Negative as well as positive features expressed in respect to expectations (e.g. seasickness, anxiety). |
| Bennett, et al., 2014, USA | 2 days | River | fly-fishing | Various psycho-social benefits identified incl. ‘reconnection’ with nature and society. |
| Vella et al., 2013, USA | 2 days, 3 nights | River | fly-fishing | Acute effects observed for improvements in attentiveness and positive mood states, coupled with significant and sustained reductions in negative mood states, anxiety, depression, and somatic symptoms of stress. |
| Ritchie et al., 2015, Canada | 10 days | Rivers, lakes | Canoe, wilderness expedition | Sensory (connecting with creation) and reflective (connecting with self) experiences made participants more aware of their surroundings and who they were as a person. |
| Ritchie et al., 2014, Canada | 10 days | Rivers, lakes | Canoe, wilderness expedition | Increased resilience in short-term (1 month). Resilience reverting back in the longterm (1 year), highlighting importance of follow-up or aftercare systems. |
| Hayhurst et al., 2015, NZ | 10 days | Sea | Sailing | Increased resilience 5 months post-intervention, social effectiveness, self-efficacy and less positive perceptions of the weather. No effect emerged for non-voyage participants. |
| Grocott and Hunter, 2009, NZ | 10 days | Sea | Sailing | Sustained increases in self-esteem post-intervention |
| Matos et al., 2007, Portugal | 4 weeks  (8 sessions) | Sea, beach | Surfing | Positive psycho-social effects - exploration, effort and perseverance, problem-solving, time management, social competencies, interpersonal relationships and emotional regulation improved during intervention |
| Fleischmann et al. 2011, USA | 6 months (1 x wk) | Sea | Surfing | Vestibular balance enhancement, pain resolution (reduced use of narcotics), Depressive symptoms resolved, temporal relief from injury and stress reduction on surf days. Impossible to determine causality from single case. |
