# Supplementary material for: Blue care: a systematic review of blue space interventions for health and wellbeing
Source: Health Promot Int. 2018 Dec 18;35(1):50–69. doi: 10.1093/heapro/day103 (PMC7245048; doi:10.1093/heapro/day103)
Supplement: day103_Supplementary_Data [file day103_supplementary_data.zip › day103-Suppl_data/Supplementary_Appendix 1.docx]

**Appendix 1:** Example of search terms used in the literature search to identify relevant literature, terms were searched for alone or in combination with AND or OR

| **Category** | **Search Terms** |
| --- | --- |
| Health/wellbeing | stress, obesity, cardiovascular, neuroendocrine, musculoskeletal change, changes in emotional states / mood (e.g., increased happiness, decreased anger), changes in cognitive capability, mental health (e.g. depression, anxiety, stress, addiction, Autism, ADHD, PTSD, etc.),disease states, disabilities (e.g. physical, spinal cord, impairment) measures of mortality, wellbeing (e.g. subjective, psychological, social, relational, quality of life), self- concept, -worth, -efficacy, resilience |
| Interaction/intervention/outcomes | intervention, restore, restorative, attain, recovery, contact, (hydro-/aquatic-) therapy, therapeutic, adventure, walking, running, exercising, stress, self-reported / perceived, swimming, surfing, boating/sailing, kayaking, white-water rafting, fly-fishing, snorkeling, diving, canoeing, rowing |
| Life-course events | birth, pregnancy, childhood, adolescence, adults, elderly, ageing |
| Study type and design effectiveness | evidence-base, review, analysis, control group, methodology,  intervention, outcome, practice, policy |
| Behavioural practices | environmental enhancement, marine conservation, proenvironmental behaviour, environmental awareness, social connection, prosocial, antisocial (behaviour) |
| Wildcards | Wildcards inter alia, effectiv*, analys*, coast* natur*, park*, exercise* |
