# Supplementary figures and images for: Blue care: a systematic review of blue space interventions for health and wellbeing
Source: Health Promot Int. 2018 Dec 18;35(1):50–69. doi: 10.1093/heapro/day103 (PMC7245048; doi:10.1093/heapro/day103)

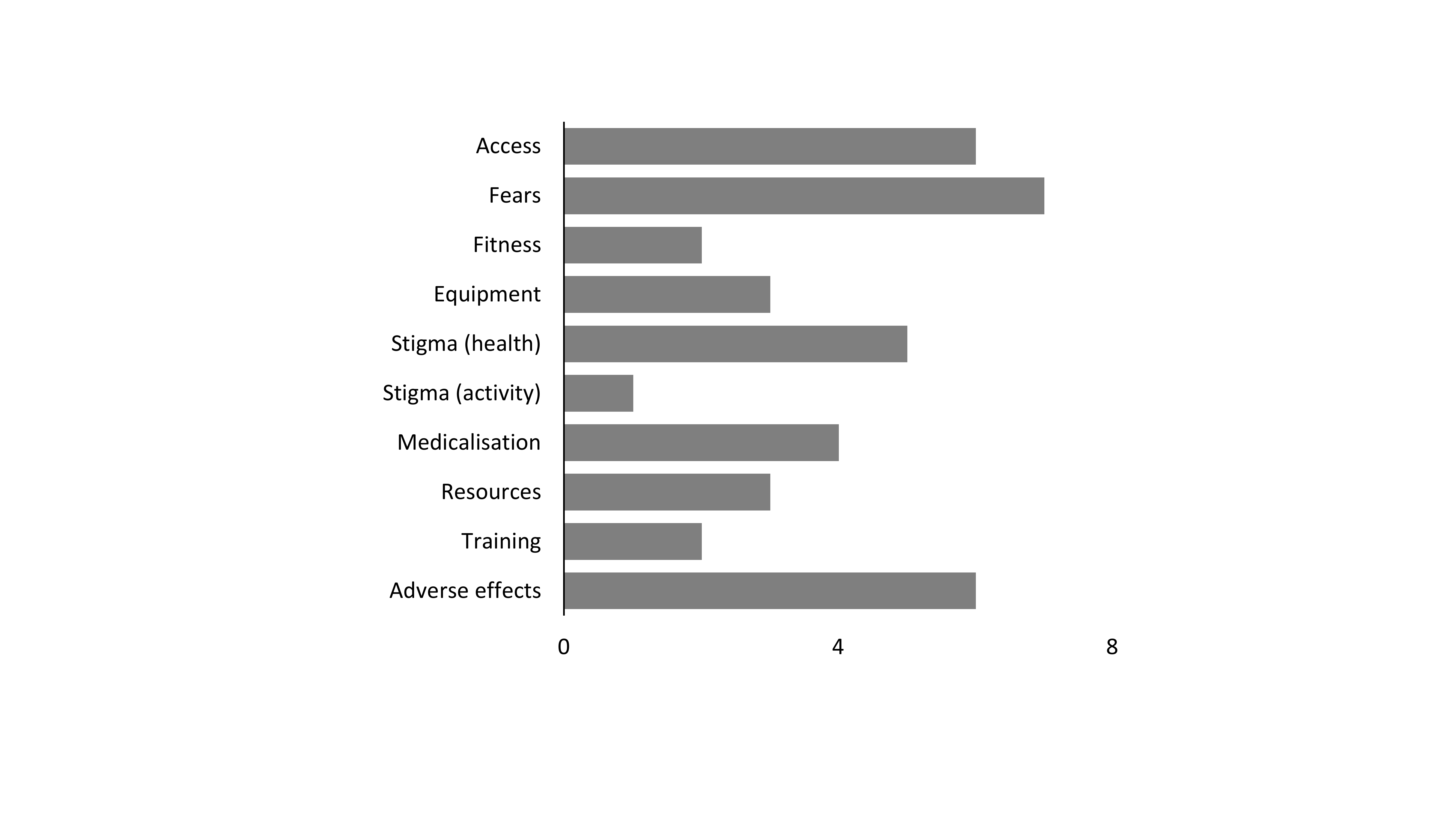

Supplement: day103_Supplementary_Data [file day103_supplementary_data.zip › day103-Suppl_data/Supplementary_Appendix 4.jpg]
